# Sterility1Abstract of a post-graduate lecture, delivered at Charing Cross Hospital on June 3, 1897.

**Published:** 1897-08

**Authors:** Amand Routh

**Affiliations:** Obstetric physician with care of out-patients at Charing Cross Hospital, and lecturer on practical obstetrics at the Medical School; physician to Samaritan Free Hospital for Women


### Selection.

### STERILITY.[1]

By AMAND ROUTH, M. D., B. S.,
Obstetric physician with care of out-patients at Charing Cross Hospital, and lecturer on practical obstetrics at the Medical School; physician to Samaritan Free Hospital for Women.

THE subject of sterility has been differently classified. In this lecture I propose very briefly to enumerate the causes of sterility resulting from abnormalities or disease in the male, and then to discuss the causes of sterility in women, omitting all cases of non-absolute sterility where conception may occur, yet a viable child is not obtained.

To fully explain my meaning I give a classification of sterility which appears to me to cover all the ground :

1. Abstract of a post-graduate lecture, delivered at Charing Cross Hospital on June 3, 1897.

CLASSIFICATION OF CAUSES OF STERILITY.

I. *Absolute, i. e.*, where there is no evidence of conception.

(*a*) Congenital—due to congenital conditions which have always existed. (*b*) Acquired—1. Primary—due to inflammation or other causes present at marriage, entirely preventing conception. 2. Secondary—coming on after the birth of a living child at same or previous marriage.

II. *Nonabsolute, i. e.*, conception takes place, yet a viable child cannot be obtained, owing to fetal death and abortion, and the like. This is usually due to noncongenital causes—acquired—and may, of course, be either primary, no fetus reaching a viable age, or secondary, due to recurring abortion due to subinvolution, and the like, after a viable birth.

I shall here only deal with causes of absolute sterility.

One in ten marriages is sterile according to Matthews Duncan. He considered the first birth usually occurs twelve to fifteen months after marriage; but may be as late as three years. Fertility in women is greatest between 26 and 38 years of age.

Medical men are often consulted by women with a view to their having children. In any such case it is better, if opportunity offers, to make enquiries as to whether the fault may not be on the husband's side. This is easily done if the patient is consulting the family doctor, but not so easy when a gynecologist is first consulted, for the husband is usually not available for the purpose. In such a case the family doctor would naturally be the one to make the necessary investigation.

In order, however, to make the subject a complete one, I will first name some of the commoner causes of sterility in the male, constituting (Kehler) probably 25 per cent. (Gervis, 7 to 15 per cent.,) of all the causes of sterility.

Causes of sterility in the male :

(1) *Constitutional*—Syphilis; Bright's disease, especially the granular kidney ; diabetes ; and lead-poisoning.

All these act by causing the spermatozoa to lack vitality, rendering them unable, therefore, to perform their essential spontaneous movements satisfactorily. These causes lead more particularly to an early death *in utero* (nonabsolute sterility), but may induce a condition of *absolute* sterility.

(2) *Local causes*—Absence of testes. Both testes undescended (cryptorchis). Double obstructive epididymitis (gonorrheal especially). Double tubercular epididymitis. Stricture of the urethra. Nonvitality, or absence of spermatozoa, as discovered by the microscope.

Impotency—which means incapacity for copulation, as opposed to sterility, which means incapacity for procreation. Although impotence is often a cause of sterility, it is not necessarily so, for conception may take place without penetration.

It is hardly necessary to add that a sterile male is not, in the preceding cases, usually impotent.

The majority of cases of sterility are, however, of female origin. Kehler says 75 per cent., Gervis 85 per cent.

Before considering the causes in the female, a few words as to the details of conception may be stated.

The semen having obtained entrance into the vagina, the spermatozoon passes by its own spontaniety upward along the genital tract, towards the ovary, against the current caused by the cilia, to encounter the ovum. During ovulation the ovum escapes from the matured and ruptured Graafian follicle, and is conveyed into the Fallopian tube and into the uterus by means of the current, which is continuously present, running from the peritoneum to the uterus, and which is caused by the action of the cilia in the Fallopian tubes. As soon as the ovum is received into the Fallopian tube it is conveyed downward by means of the cilia. Contact between the spermatozoa and ovum may ensure impregnation at any part of this transit. During the passage along the tube, the mucous membrane of the uterus is being prepared for the reception of the ovum by the formation of the decidua vera. If any of these processes are absent or interfered with, sterility may result.

Bearing this in mind, therefore, the following are groups of causes which would occur to one during the examination of any given case :—

(1) *Causes which prevent penetration* have to be dealt with first. It must, however, be remembered that in exceptional cases of impotence, or after intentional "withdrawal" where intravaginal emission has not occurred, conception may yet take place. Amongst these causes are some congenital ones: absent or solid vagina; stenosis of the vagina ; imperforate or resistant hymen.

Some of these vaginal conditions may be simulated by inflammatory processes ; and they may, with greater or less difficulty, be dealt with by operation, and the sterility may then sometimes be cured. They are at once ascertained by inspection and digital examination. If the uterus and ovaries are present and well developed, the history would show that amenorrhea, with dysmenorrhea, had existed since puberty, and there would perhaps be a hypogastric swelling, due to either hematometra, or to elevation of the uterus by hematocolpos. Vaginismus and dyspareunia are causes occasionally preventing penetration, and may be due to either a local state alone, or superadded to a state of general neurosis.

(2) *Penetration may be normal, yet the spermatozoa may not be able to ascend* beyond the upper end of the *vagina*, or if they do ascend, no suitable nidus *in utero* is available.

Amongst the causes relating to this group may be mentioned:

Absent or solid or very undeveloped uterus (incurable).

Immature uterus. This may tardily but sufficiently mature.

The so-called sterile uterus, which is really a small uterus associated with anteflexion, a condition normal during infancy, with a conical and often elongated cervix, and round instead of transverse external os uteri.

Cervical stenosis, congenital or acquired, with perhaps resulting hematometra (if complete). This might be curable by dilatation.

Hyperinvolution.

Post-climacteric atrophy.

Destruction of lining membrane by atrophic endometritis, or by use of escharotics, such as nitric acid.

Fallopian tubes, solid or absent (not diagnosable).

Tubal stenosis, or constriction of the tubes by external adhesions, or closure of the peritoneal end of the tube by lymph.

Tubes distended by serum, pus or blood (hydro-, pyo-, or hemato-salpinx).

Most of these would be determined by bimanual examination with, or without, the use of the sound.

(3) Conditions involving *destruction or loss of vitality of the spermatozoa during transit* along the genital tract. These are mainly inflammatory states, especially those due to gonorrhea, affecting the vagina, endocervix, endometrium, or lining membrane of Fallopian tubes, producing acrid discharges. Subinvolution is only a cause because it is often associated with endometritis. Fungous endometritis is also a common cause of sterility, and is often a pure overgrowth, not of inflammatory origin at all.

(4) The *passage from the vulva to the fimbriated extremity of the tube may be quite patent and there may be no inflammation present*, yet sterility is present. The reason may be *in the function*

*of ovulation.* Either the ovary is absent or contains no follicles ; or the ovary has become completely sclerosed and its follicles destroyed by inflammatory or malignant changes. If the ovaries were congenitally absent or functionless, there would be moderate anemia, with absence of all menstrual molimina, and there would almost certainly be absence of the signs of puberty. Occasionally double ovarian cysts may lead to complete destruction of the epioöphoric elements. Double ovariotomy or removal of both appendages would, of course, lead to the same result. One of the commonest causes, however, of faulty ovulation is a thickened capsule, the result of perioöphoritis. This prevents the escape of the matured ovum, and in turn leads to retention cysts being formed, and secondarily to disorganisation of the neighboring stroma and follicles.

(5) *Ovulation itself may be normal, yet the ovum is not received into the Fallopian tube.* This may be due to blocking of the tube near or at its fimbriated end, or to other tubal obstructive disease, such as hydro- or pyo-salpinx ; or the mischief may be due to an altered position of the ovary, especially if it is fixed at some distance from its tube.

If there has been salpingitis, the tubal cilia are destroyed, and the current from the peritoneum to the uterus is absent.

I have said very little as regards uterine displacements, mainly because they do not *per se* produce sterility. An anteversion or an anteflexion would never produce sterility, unless associated with a true narrowing of the external or internal os uteri, or with other developmental immaturity, or with some inflammatory state. Retroversion and retroflexion, however, are much more likely to cause sterility, which is, as a clinical fact, often cured by replacement and a well-fitting Hodge ; but even then the sterility is more often directly due to a coëxistent endometritis, or endocervicitis, than to the mere displacement. Fibroids again, though often productive of sterility, often lead rather to very early abortion ; and even if sterility does exist, it is often due to a coëxisting fungous endometritis, or a cervical catarrh, or to the menorrhagia lasting so long that the duration of the process of ovulation is entirely covered by the menstrual loss. Or there may result a mechanical torsion of the canal, or a dragging up of the cervix, or perhaps even more frequently fibroids tend to displacement, torsion, or stretching of the Fallopian tubes. Malignant disease is also a frequent cause of acquired sterility ; but, coming on late in life, may practically be said to be rarely a cause of primary sterility. It is not very rare also for pregnancy to coëxist with epithelioma of the cervix.

*Constitutional causes* of sterility in women are mainly those conditions which lead to impaired nutrition ; but even here the sexual organs seem to be capable of carrying on their functions when all other organs have their functions impaired or in abeyance. Even constitutional syphilis probably acts mainly in this way by impairing nutrition.

One must not forget to allude to *causes of apparent sterility*, due to "preventive" methods being adopted by the consent of both parties, or even by husband, or wife, unknown to his or her partner. Dyspareunia and vaginismus again may lead to sterility by preventing marital intercourse altogether.

The examination of a sterile woman with a view to discovering the cause should be conducted methodically, after the necessary questions have been put as to dyspareunia, and the like. The breasts should be examined to note the presence or absence of the mammary glands and the development of the nipples. Lack of development here is often a fair indication of a similar condition of the pelvic organs. The abdomen should then be examined with a view to discovering the presence of an abdominal tumor, as would obtain in fibroids, or hematometra, due to occlusion. At the same time it will be noticed whether the pubes is normally developed. Inspection of the vulva and digital examination of the vagina will be made, and any spasm, constriction or stenosis noted. By the same means the cervix would be examined and such details as a conical shape, or small os, discovered. By bimanual examination the size, shape, outline, consistence and mobility of the uterus would be determined, and the presence of any periuterine swelling in the posterior quarters of the pelvis or in Douglas's pouch would also be felt. The relations of these to the uterus, with their relative mobility, their consistence, and their degrees of tenderness, would help one greatly to determine whether they were uterine, ovarian or tubal, or were perimetric inflammatory exudations. The sound should rarely be used, but may be useful to confirm the results of a bimanual examination, and the speculum may also occasionally be usefully employed.

Successful treatment of sterility depends mainly upon the removal of the causes. Sometimes this is exceedingly easy, sometimes it is impossible. It is easy, for instance, in the curing of an inflammatory state, in the dilatation of a narrow uterine canal, as may occur in cases associated with spasmodic dysmenorrhea. Treatment is easy also in cases of retroversion, where replacement can be easily effected. Treatment is difficult where the ovaries or tubes are at fault, the result of displacement or of inflammation; and successful treatment is practically impossible when the cause is lack of development in the organs concerned, or where fibroids have formed, or malignant disease is present.

In any case where it is not absolutely certain that the sterility is due to causes in the woman under consideration, it is far better to see the husband, or to get him to see some surgeon skilled in such cases, so that any causes of sterility on his part may be discovered and an unnecessary operation on the wife avoided. Much tact must be exercised in such an examination of the husband, for it unfortunately happens that he will rarely admit the possibility of the fault being on his side, and almost invariably assumes that his partner is the one in fault.

This probably arises from the fact that the sexual appetite may be absent in the wife, and the husband's assumption is the result of a popular, but erroneous belief, that the presence of such satisfaction is essential to procreation.—*Treatment.*

---

The Duration of Infection in Whooping-Cough.—Weill, who in 1894 expressed the opinion that whooping-cough is contagious only during the premonitory catarrhal stage, has since put his opinion to the test. On various occasions he permitted nearly one hundred young children, who had not previously suffered from whooping-cough, to be associated in the same ward, for twenty days or more, with children suffering from the disease during the stage of whooping. In only one case was the disease contracted, and in this instance the patient, from whom the infection was derived, was in the very earliest period of the whooping stage. In three small epidemics Weill was able to satisfy himself that infection was contracted from children who had not yet begun to whoop. He concludes that infection ceases very soon after the characteristic whoop commences and that, therefore, in a family it is not the patient who is already whooping, but his brothers and sisters who have not previously had whooping-cough, who ought to be isolated.—*Lyon Méd.*, May 19, 1897.—*Universal Medical Journal.*

